# Clinical Application of Skeletal Muscle Quantity and Quality Assessment Using Bioelectrical Impedance and Ultrasound Images

**DOI:** 10.1298/ptr.R0031

**Published:** 2024-07-05

**Authors:** Masashi TANIGUCHI

**Affiliations:** ^1^Human Health Sciences, Graduate School of Medicine, Kyoto University, Japan

**Keywords:** Muscle mass, Intramuscular fat, Muscle degeneration, Echo intensity, Extracellular-to-intracellular water ratio

## Abstract

A decline in muscle strength is a key factor responsible for physical dysfunction in older individuals. Both loss of muscle quantity and quality are associated with muscle strength decline. While the gold standard method for evaluating muscle mass and quality is magnetic resonance imaging, it is not suitable for clinical settings because of the measurement and analysis costs. Bioelectrical impedance analysis (BIA) and B-mode ultrasonography are clinically useful alternatives for skeletal muscle assessment owing to their feasibility and noninvasiveness. The recent advancements in the techniques for BIA and ultrasonography have improved their accuracy in assessing skeletal muscle quantity and quality, making them useful in detecting age-related and disease-specific alterations. This review comprehensively analyzes the advantages of using BIA and ultrasound imaging for assessing skeletal muscle quantity and quality and detecting muscle degeneration. We summarize the recent findings regarding age-related changes in muscle characteristics and the associations of muscle degeneration with physical dysfunction in patients with knee osteoarthritis. Furthermore, we discuss the clinical application of skeletal muscle assessment using BIA and ultrasound for evaluating training effects and exercise prescription.

## Introduction

Skeletal muscle tissue is a large tissue in the human body with remarkable plasticity. However, a decline in muscle function is associated with functional disability in daily activities, such as walking and sports. Physiologically, age- and disease-related decline in muscle function is reflected as muscle atrophy, that is, a reduction of muscle mass as well as muscle quality, which is replaced by the accumulation of intramuscular fat (intraMAT) or other noncontractile tissues. Typically, magnetic resonance imaging (MRI) is recommended for precise muscle mass evaluation and assessment of intraMAT infiltration based on differences in signal intensity. Clinically, muscle function can be objectively quantified using relatively inexpensive and noninvasive techniques, such as bioelectrical impedance analysis (BIA) and B-mode ultrasound imaging. Lately, remarkable developments have been made in terms of performance improvement and miniaturization of these devices which enables convenient evaluation of muscle function in the clinical setting. The current review presents an overview of the techniques used for assessing skeletal muscle quantity and quality, including the standard MRI and alternative methods such as BIA and ultrasonography, and outlines their characteristics to offer insights into the clinical applications and future research in this field.

## Significance and Concerns Regarding Muscle Quantity and Quality Assessment

The age-related loss of muscle mass, strength, and physical function is called sarcopenia^1)^. Since sarcopenia is a potential risk factor for reduced mobility and increased falls and mortality, evaluating muscle function is crucial for all older individuals, especially in a rapidly aging society, regardless of their health status. Muscle mass gradually diminishes from the late 40s^2)^, with the decline accelerating with aging. MRI and computed tomography (CT) are considered the gold standards for accurately evaluating muscle mass and cross-sectional area (CSA). In addition to the age-related decrease and atrophy of muscle fibers (muscle cells), there is a concomitant increase in intramuscular noncontractile elements, such as ectopic fat, fibrous connective tissue, and extracellular water in the intercellular space^3)^. These changes are representative of a loss of muscle quality and are a risk factor for future fractures and mortality because an increase in noncontractile elements hinders muscle force production.

**Fig. 1.**
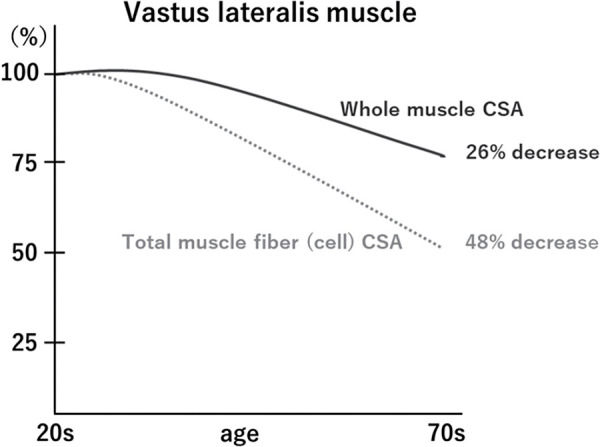
Age-related decrease in whole muscle cross-sectional area and total muscle fiber area^5)^ The decrease in total muscle fiber CSA (number of muscle fibers × mean fiber size) represents a more remarkable age-related change compared to the decrease in whole muscle CSA, which indicates an underestimation of actual muscle atrophy and highlights the significance of identifying noncontractile elements from whole muscle CSA. CSA, cross-sectional area

Evaluating muscle CSA using MRI and CT and muscle thickness (MT) using ultrasound also includes the noncontractile elements in the intercellular space, which risks masking the actual muscle atrophy^4)^. Yamada^5)^ verified the age-related decline in whole muscle CSA and total muscle fiber CSA (number of muscle fibers × mean muscle fiber size) reported earlier by Lexell et al.^3)^ and found that the whole muscle CSA decreased by 26% and the total muscle fiber CSA by 48% in old compared to young subjects (Fig. 1). This implies that there is a relative reduction in the myofiber area within the total CSA of the muscle with age, which is often seen in the underestimation of actual muscle atrophy because of the expansion of intercellular spaces. Therefore, quantification of qualitative changes, particularly focusing on noncontractile elements, is essential for truly assessing muscle degeneration and is a topic of great interest in contemporary skeletal muscle research.

Differences in signal intensity on MRI are used to judge qualitative changes in skeletal muscle. Although it is an excellent tool for quantification, it lacks feasibility because of the associated difficulties with measurement and analysis costs. By contrast, BIA and ultrasonography are effective, low-cost, noninvasive, and portable alternatives for evaluating qualitative changes in muscle quantity and quality, especially as screening tools in clinical settings where MRI or CT cannot be conducted. The subsequent sections explain the use of MRI, BIA, and ultrasound for qualitatively assessing muscle degenerative changes.

## MRI: The Gold Standard for Evaluating Muscle Mass and Quality

**Fig. 2.**
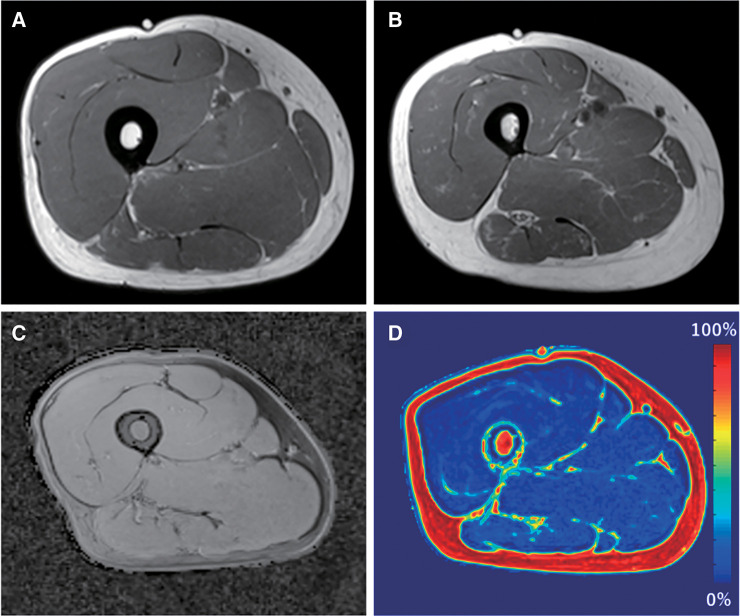
Muscle quantity and quality assessment using MRI Representative T1-weighted MR images of the thigh muscles of a healthy young man (A) and an older man (B) used for assessing the muscle CSA. Subcutaneous fat deposition within the thighs is more pronounced in older males compared to younger males, alongside observable signs of muscle atrophy. In addition, B exhibits an increase in intramuscular high-intensity areas, indicative of intraMAT. (C) A composite image of the in-phase and out-phase was obtained using the Dixon method. (D) A color map to quantify intraMAT is determined by the water-to-fat ratio; a higher ratio of intraMAT (the redder the color map) indicates increased intraMAT. CSA, cross-sectional area; intraMAT, intramuscular fat infiltration

In MRI, the T1 relaxation time is shorter in adipose tissue and relatively longer in muscle tissue; therefore, adipose tissue displays higher signal intensity (white), and muscle tissue shows lower signal intensity (black) in T1-weighted images (Fig. 2A, 2B). Using these differences in signal intensity, adipose tissue can be distinguished from muscle tissue in the MR image, allowing for the measurement of its proportion in the muscle CSA. Briefly, the procedure for fat quantification on T1-weighted images is as follows: we establish a region of interest (ROI) on the T1-weighted image which contains equal areas of subcutaneous fat and muscle. The adipose tissue is distinguished from muscle tissue using a threshold value derived from the histogram of pixel numbers and signal intensity within the ROI. In a previous study investigating intraMAT infiltration in thigh muscles, the threshold value for distinguishing adipose tissue from muscle tissue was calculated using the “Otsu threshold method” from histograms drawn with each ROI set for the vastus intermedius muscle and subcutaneous fat^6)^.

The Dixon method is the standard analytical approach used for assessing intraMAT; it utilizes the differences in resonance frequencies between fat and water protons (Fig. 2C, 2D). Fat and water protons rotate at slightly different frequencies (difference = 3.5 ppm), because of which fat and water protons point in the same direction (in-phase) at echo times (TE) of 0, 4.5, and 9 ms in the case of 1.5T, and in the opposite directions (out-phase) at a TE of 2.25 and 6.75 ms. The Dixon method utilizes the signal intensity of each pixel in the in-phase and out-phase images to differentiate between fat and water, thereby generating separate fat and water images. Quantification of intraMAT (in percentage) is computed as the signal intensity of the fat image relative to the sum of the signal intensities of the fat image and water image. Unlike the assessment of intraMAT using T1-weighted images, the Dixon method eliminates the need for calculating a threshold value^7)^. Both methods, using T1-weighted images and the Dixon technique, have been validated against fat fraction obtained from muscle biopsy^8^^,^^9)^.

## Bioelectrical Impedance Analysis

BIA evaluates body composition and estimates the proportion of different body tissues by measuring the differences in specific resistance (impedance) of body tissues when an alternating current passes through the body. It is a cost-effective, compact, and rapidly deployable quantification technique. BIA is used to estimate the skeletal muscle mass index (SMI), which is calculated as the appendicular skeletal muscle mass divided by the square of height. SMI is used to quantify muscle mass for diagnosing sarcopenia; the cutoff values of 7.0 kg/m^2^ for men and 5.7 kg/m^2^ for women are used for defining the presence or absence of muscle mass decline^1)^.

Conventional BIA mainly employs a single frequency of 50 kHz, which has the disadvantage of susceptibility to peripheral water content, excessive exercise, and postural changes. As a result, multifrequency BIA (employing both low and high-frequency currents) is used nowadays. It differentiates between the water content within muscle cells (intracellular water; ICW) and outside muscle cells (extracellular water; ECW) based on the electrical properties that low-frequency current cannot pass through the muscle cell membrane while high-frequency current can.

**Fig. 3.**
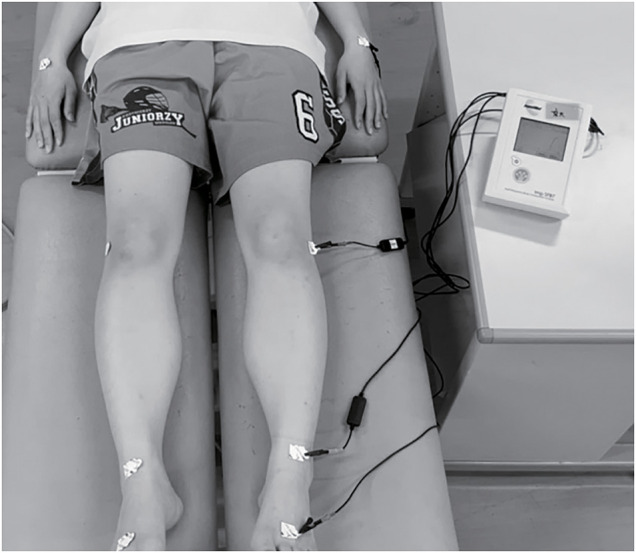
Measurement of the bioelectrical impedance using S-BIS The image shows the setting for S-BIS measurement in the lower leg. The participant rests in a relaxed position to avoid body water redistribution. Two sensing electrodes and two injection electrodes are placed on landmarks on the body surface to obtain the bioelectrical impedance, The values so obtained are input into the estimation algorithm to calculate ECW and ICW^37)^. S-BIS, segmental bioelectrical impedance spectroscopy; ECW, extracellular water; ICW, intracellular water

Recently, segmental bioelectrical impedance spectroscopy (S-BIS) has also been applied, which has higher theoretical accuracy and the ability to evaluate arbitrary regions (Fig. 3). In both BIA and S-BIS, the ECW/ICW ratio, which is calculated as the ECW divided by the ICW, is used as an indicator of qualitative changes in muscle which can be used for evaluating physical function. A higher ECW/ICW ratio implies a relative increase in the proportion of non-contractile elements to the contractile elements, indicating loss of muscle quality. Previous studies have shown that the ECW/ICW ratio increases with age, and correlates with muscle strength and walking ability independent of muscle mass^10^^,^^11)^.

## B-mode Ultrasound

**Fig. 4.**
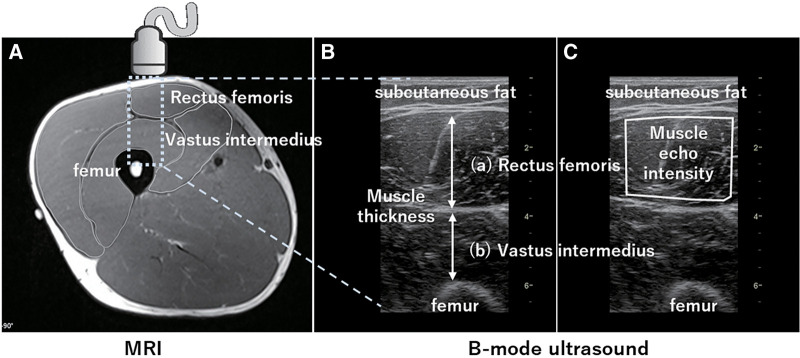
The measurements of muscle thickness and echo intensity using B-mode ultrasound (A) An axial MRI captured at the midthigh region. (B) A B-mode image taken at the same site. In this B-mode image, MT of (a) rectus femoris is measured as the distance between the muscle fascia, and MT of (b) vastus intermedius muscles is measured as the distance between the muscle fascia and bone interface. (C) The ROI for obtaining EI is set to avoid the surrounding fascia and the average EI within the ROI is calculated using image analysis software. A higher EI value (represented by white areas) indicates augmented fat deposition within the muscle. MT, muscle thickness; ROI, region of interest; EI, echo intensity

B-mode ultrasound generates images of the body tissues using echoes of an ultrasound beam passing through body tissues when a probe is in contact with the skin. The MT measured by an ultrasound image shows a moderate to strong positive correlation with the muscle volume and CSA measured by MRI^12)^, and is clinically used as an indicator of muscle mass (Figs. 4A, 4B). In addition, muscle echo intensity (EI), represented as grayscale in an ultrasound image, is an established indicator of qualitative muscle changes. Muscles with increased adipose and fibrous tissue have a whitish appearance on ultrasound images (i.e., higher EI), suggesting a decrease in muscle quality^13)^. EI is measured in 256 gradations (8-bit grayscale), where black is 0 and white is 255, and is calculated as the average value within an ROI (Fig. 4C). Analysis of EI using 8-bit grayscale is extremely feasible with a variety of image analysis software, many of which are freely available. EI is associated with muscle weakness in older individuals^14)^ and is significantly elevated in conditions such as lower-limb osteoarthritis (OA)^15^^,^^16)^ and neuromuscular disorders^17)^. A moderate correlation has been reported between EI and intraMAT fraction measured by the Dixon method^18^^,^^19)^.

Similar to BIA, B-mode ultrasound can be conducted rapidly and is noninvasive and economical. Furthermore, it offers the advantage of real-time evaluation of individual muscles and can be used for visual feedback during muscle contraction. Conversely, it requires skilled personnel for probe operation and it is not possible to assess the entire limb or whole body at the same time. It is noteworthy that (1) there is no clear threshold EI value as it varies depending on the ultrasound equipment and settings and (2) it is less adaptable for deep muscles because the echoes attenuate with increasing tissue depth.

## Clinical Assessment of Muscle Quantity and Quality Using BIA and Ultrasound

### Advantages/disadvantages of BIA and ultrasound images for clinical assessment

BIA is suitable for screening muscle quantity and quality in the extremities because it can easily assess body composition in a short time; however, it cannot be used to evaluate individual muscles. BIA is applicable in most individuals unless they have an implanted digital device, such as a cardiac pacemaker. During periods of significant fluctuations in body fluids, such as fluid infusion or evident edema, bioimpedance values may not be accurate, necessitating a cautious interpretation of the assessment results.

By contrast, B-mode ultrasonography can be easily used to assess individual muscles and detect muscle-specific changes; however, accurate evaluation of muscles requires a thorough understanding of ultrasound settings and operational techniques.

Therefore, both techniques can be used to obtain a more accurate assessment of changes in muscle quantity and quality. Understanding the advantages and disadvantages of each technique is essential to select the appropriate device based on specific evaluation objectives.

### Age-related muscle degeneration

In age-related skeletal muscle degeneration, enhanced EI occurs at an earlier stage than a decrease in MT. A previous study indicated that EIs in biceps brachii, quadriceps, and transversus abdominis muscles are higher in middle-aged than in young, while MTs were not significantly different between groups^20)^. This means there is a substantial decrease in muscle fiber mass even in the absence of an obvious decrease in MT because of the increase in intraMAT infiltration and fibrosis within the interstitial space between muscle fibers.

**Fig. 5.**
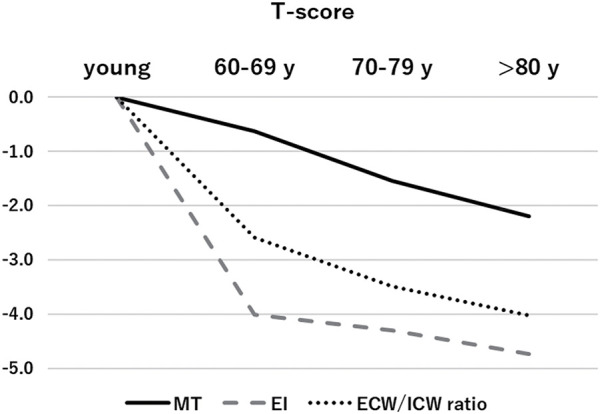
Age-related changes in MT, EI, and ECW/ICW ratio The figure shows the changes in T-scores for the 60s, 70s, and 80s age groups compared to the young group. The decline in T-score was most notable in the order of EI > ECW/ICW ratio > MT. While all muscle degeneration indicators are influenced by age, the pattern of decline varies among these indicators. MT, muscle thickness; EI, echo intensity, ECW, extracellular water; ICW, intracellular water

Studies assessing muscle degeneration in older individuals compared to young individuals indicate that EI is significantly influenced by aging in the initial phases, with its progression being gradual (Fig. 5)^21)^. A previous study tracked longitudinal changes in quadriceps MT and EI over 4 years in 131 healthy older individuals and found that quadriceps MT significantly decreased by 11.5% during this time while, interestingly, EI showed no significant change^22)^. Thus, a decline in muscle quality significantly affects muscle function from the early stages of aging, while the effects of reduced muscle mass become more apparent at older ages. The authors also verified whether indices of muscle quality (EI and ECW/ICW ratio) were independently associated with muscle strength in community-dwelling older individuals. They reported that muscle strength was significantly and positively correlated with MT, whereas it was negatively correlated with EI and ECW/ICW ratio; notably, all indices were independently associated with muscle strength^11)^. In addition, a weak positive correlation exists between EI and ECW/ICW. These findings indicate that the indices EI and ECW/ICW ratio complement each other in assessing qualitative muscle changes that may not be fully captured by either alone.

For noncontractile elements, enhanced EI primarily reflects the infiltration of fat tissue, but enhanced EI does not reflect water content. Whereas, an increased ECW/ICW ratio reflects the increase in ECW, including that in fat tissue. Therefore, both EI and the ECW/ICW ratio are indicators of muscle quality associated with muscle weakness in older individuals, but each index assesses different aspects of muscle quality.

### Disease-specific muscle degeneration in knee OA

Atrophy of the quadriceps femoris muscle is a well-known factor associated with muscle weakness in patients with knee OA. A recent systematic review revealed a significant increase in noncontractile elements within the quadriceps femoris muscle among patients with knee OA^23)^, highlighting the influence of muscle quality on muscle weakness and functional disability. This was confirmed in muscle biopsies of patients with knee OA; muscle weakness was associated with increased deposition of excess collagen between muscle fibers but not muscle fiber atrophy^24)^. Furthermore, the increase in intramuscular noncontractile elements in knee OA was relatively larger than that anticipated with age-related changes. A study identified 787 patients with bilateral knee OA from a large-scale prospective cohort and examined the association of muscle quantity (lower extremity SMI) and quality (ECW/ICW ratio) obtained through multifrequency BIA on functional decline^25)^. Interestingly, higher ECW/ICW, and not lower extremity SMI, was associated with physical dysfunction in knee OA, with greater association in patients with severe symptoms. In addition, higher EI on B-mode ultrasonography of the vastus medialis muscle was observed in patients with mild-to-severe OA (Kellgren-Lawrence; KL grade >2)^15)^ and was deemed a predictor of future functional decline and worsening symptoms^26)^. Increased intraMAT in the quadriceps, particularly in vastus medialis, has been detected even in the early stages of knee OA (KL grade <2), suggesting that deterioration of muscle quality occurs before the onset of knee OA^27)^.

**Fig. 6.**
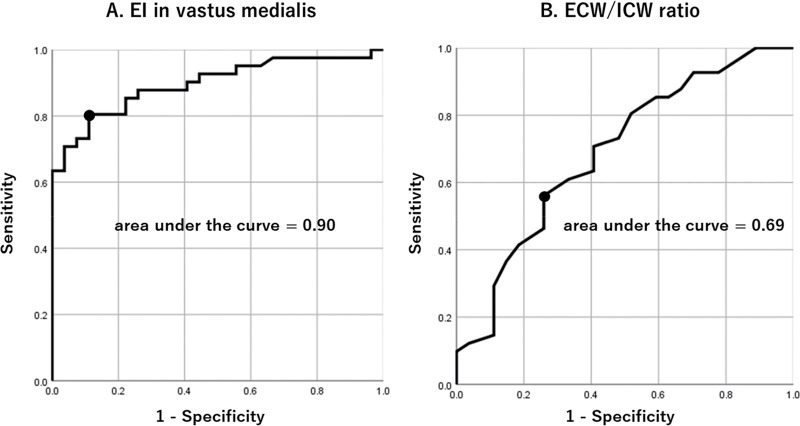
ROC analyses for detecting muscle degeneration characteristics with knee OA^28)^ ROC curve analyses were conducted with the knee OA group (reference, healthy control group) as the dependent variable. (A and B) The results of the ROC analysis on EI in the vastus medialis and ECW/ICW ratio, respectively. The area under the curve value was higher in EI of the vastus medialis than in ECW/ICW, suggesting a higher discrimination accuracy of EI in identifying degeneration in the vastus medialis in patients with knee OA. ROC, Receiver-operating characteristic curve; OA, osteoarthritis; EI, echo intensity. ECW, extracellular water; ICW, intracellular water

A higher ECW/ICW ratio and enhanced EI of the vastus medialis are helpful clinical signs for detecting muscle degeneration in patients with knee OA. However, it is noteworthy that vastus medialis EI has a higher discrimination accuracy for characterizing muscle degeneration in knee OA than the ECW/ICW ratio^28)^. This may be because EI measurement using ultrasound assesses an individual muscle, such as the vastus medialis, whereas the ECW/ICW ratio measured using S-BIS cannot distinguish between the four individual muscles of the quadriceps (Fig. 6). Hence, both EI and the ECW/ICW ratio are useful biomarkers for predicting functional decline and symptom severity in patients with knee OA.

## Application to the Index of Strength Training Effectiveness

### Indicators of muscle hypertrophy

Changes in muscle volume and CSA are used for evaluating muscle hypertrophy with resistance training. Since measuring muscle volume and CSA using MRI and CT is not suitable in clinical settings, multifrequency BIA is commonly used as a simple alternative to estimate muscle volume. However, conventional multifrequency BIA evaluates muscle volume in the entire limb and has limited use in assessing individual segments, such as the thigh and lower leg. Therefore, certain formulas were developed to estimate thigh and quadriceps muscle volume obtained from MRI using S-BIS (Table 1)^12)^. The accuracy of the model for estimating muscle volume in these equations can be improved by considering the ECW/ICW ratio, an index that reflects muscle quality in addition to the conventional 50 kHz impedance index. It should be noted that these equations are for muscle volume in young individuals, although considering the ECW/ICW ratio as muscle quality. The quadriceps MT index, calculated as the product of the maximum MT measured at midthigh and thigh length, is comparable in accuracy to the muscle volume estimation formula derived from S-BIS. The use of these estimation equations allows for highly accurate and localized assessment of muscle quantity using BIA and ultrasound.

**Table 1.** The estimation equations for the thigh and quadriceps muscle volume^12)^

For the thigh muscle volume (cm^3^)
Equation 1 = 45.4 × 50-kHz BI index – 581.2 × ECW/ICW index + 2,180.6
Equation 2 = 119.5 × ICW index + 927.5
For the quadriceps muscle volume (cm^3^)
Equation 1 = 78.4 × ICW index + 779.5
Equation 2 = 10.3 × muscle thickness index + 35.3

The above mentioned estimation equations for thigh and quadriceps muscle volumes have similar accuracy. The index value input into the estimation equations is calculated as follows:
The 50-kHz BI index is calculated as the thigh length^2^/Z_50_ (cm^2^/Ω). The impedance of the ICW compartment (Z_250−5_) is calculated as 1/[(1/Z_250_) − (1/Z_5_)]; then, the ICW index is calculated as the thigh length^2^/Z_250−5_ (cm^2^/Ω). The muscle thickness index (cm^2^) is calculated as the product of muscle thickness and thigh length.

BI, bioelectrical impedance; ECW, extracellular water; ICW, intracellular water

### Indices of the immediate effectiveness of resistance training

**Fig. 7.**
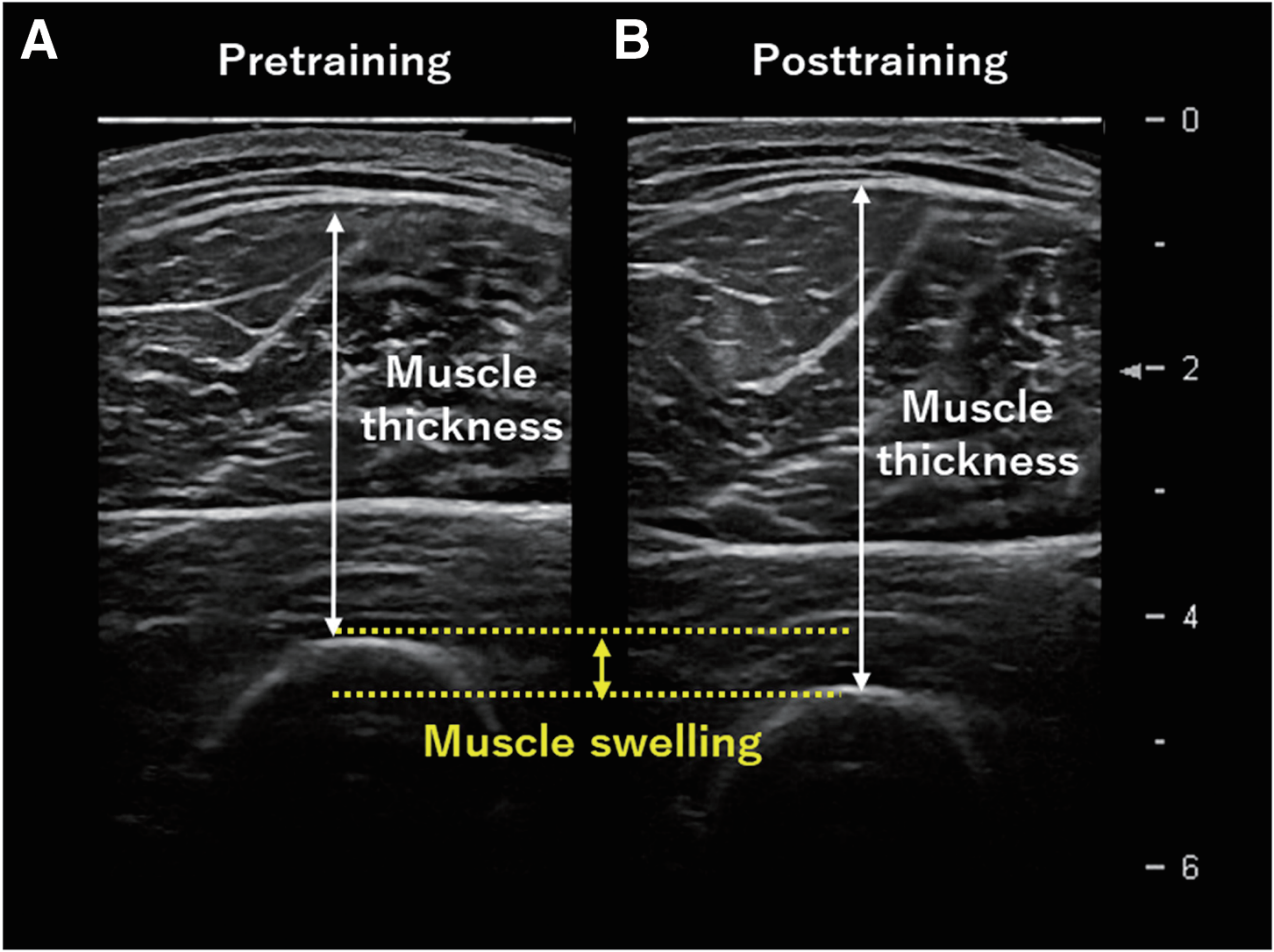
Immediate changes in MT and EI with resistance training An increase in MT can be confirmed in post-resistance training (B) compared to pre-resistance training (A) using B-mode ultrasonography. Muscle swelling, defined as the augmentation in MT following resistance training, is observed concurrently with an increase in EI, This immediate higher EI is associated with delayed onset muscle soreness. MT, muscle thickness; EI, echo intensity

**Fig. 8.**
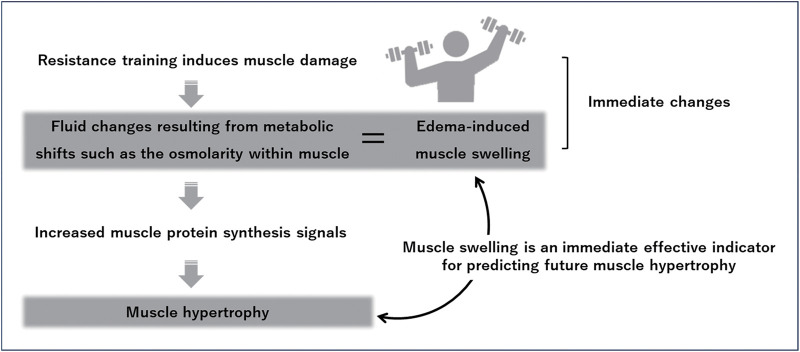
Schema of immediate and long-term effects of resistance training

S-BIS and ultrasound are useful in assessing the immediate effectiveness of resistance training. MT exhibits a temporary increase following resistance training (Fig. 7) because of “edema-induced muscle swelling,” a phenomenon that occurs due to swelling both inside and outside the muscle fibers resulting from muscle damage with resistance training^29^^,^^30)^. Muscle swelling is an important effect index observed at the beginning of the training session and served as an indirect marker of myofibrillar protein synthesis^31)^. A previous study indicated that more muscle swelling following the first session of resistance training moderately correlates with greater muscle hypertrophy after an 8-week intervention, suggesting that muscle swelling is a valid immediate-effect indicator for predicting future muscle hypertrophy (Fig. 8)^32)^. The EI also exhibits a temporary increase following resistance training. Enhanced EI immediately after resistance training reflects an inflammatory response in muscle fibers due to muscle damage and is associated with delayed onset muscle soreness^33)^. Confirming the immediate effects of resistance training by evaluating changes in MT and EI at the initial session allows for effective training prescriptions and ensures long-term intervention effects.

In addition, assessing the ECW fluctuations using S-BIS helps quantify muscle swelling. Traditionally, immediate water redistributions within the muscle following resistance training are quantified by measuring the T2 relaxation time using T2-weighted MR images^34)^. The prolongation of T2 relaxation time following resistance training is associated with alterations in the inorganic phosphate/phosphocreatine ratio and intracellular pH, reflecting fluid changes resulting from metabolic shifts such as the osmolarity of skeletal muscle^35)^. Based on this theoretical background, a study investigated the association between the ECW/ICW ratio measured using S-BIS and muscle swelling^36)^. A significant positive correlation was observed between muscle swelling induced by high-load resistance training in the quadriceps femoris and the ECW/ICW ratio. Thus, the ECW/ICW ratio measured using S-BIS can be an immediate indicator of training effectiveness. Given the noninvasiveness, feasibility, and cost-effectiveness of this method, it is a valuable alternative to MRI for assessing muscle quantity and quality and is expected to be widely used in clinical settings.

## Conclusion

This review summarizes the significance and specific methodology for assessing skeletal muscle quantity and quality using BIA and B-mode ultrasonography in clinical settings. Understanding the characteristics of BIA and ultrasonography in assessing skeletal muscle function will help enhance the accuracy of the existing techniques for detecting muscle degeneration and assessing training effects. Owing to their relative accuracy, noninvasiveness, feasibility, and cost-effectiveness, BIA and B-mode ultrasonography are expected to be used as screening tools for muscle function assessment.

## Acknowledgments

The author would like to thank Yoshihiro Fukumoto (Kansai Medical University), Yosuke Yamada (National Institutes of Biomedical Innovation, Health and Nutrition), Masahide Yagi (Kyoto University), and Noriaki Ichihashi (Kyoto University) for their comprehensive instruction on related studies.

## Funding

Not applicable.

## Conflicts of Interest

The author declares no conflicts of interest.

## References

[1] Chen LK Woo J, *et al.*: Asian working group for sarcopenia: 2019 consensus update on sarcopenia diagnosis and treatment. J Am Med Dir Assoc. 2020; 21: 300–307.e2. 32033882 10.1016/j.jamda.2019.12.012

[2] Janssen I Heymsfield SB, *et al.*: Skeletal muscle mass and distribution in 468 men and women aged 18-88 yr. J Appl Physiol (1985). 2000;89: 81–88. 10904038 10.1152/jappl.2000.89.1.81

[3] Lexell J Taylor CC, *et al.*: What is the cause of the ageing atrophy? Total number, size and proportion of different fiber types studied in whole vastus lateralis muscle from 15- to 83-year-old men. J Neurol Sci. 1988; 84: 275–294. 3379447 10.1016/0022-510x(88)90132-3

[4] Yamada Y Schoeller DA, *et al.*: Extracellular water may mask actual muscle atrophy during aging. J Gerontol A Biol Sci Med Sci. 2010; 65A: 510–516. 10.1093/gerona/glq001 20133393

[5] Yamada Y: New approach focused on muscle cell mass and muscle composition for the definition of skeletal muscle mass and sarcopenia. Jpn J Phys Fit Sports Med. 2015; 64: 461–472. (in Japanese)

[6] Akima H Yoshiko A, *et al.*: Skeletal muscle size is a major predictor of intramuscular fat content regardless of age. Eur J Appl Physiol. 2015; 115: 1627–1635. 25757882 10.1007/s00421-015-3148-2

[7] Alizai H Nardo L, *et al.*: Comparison of clinical semi-quantitative assessment of muscle fat infiltration with quantitative assessment using chemical shift-based water/fat separation in MR studies of the calf of post-menopausal women. Eur Radiol. 2012; 22: 1592–1600. 22411305 10.1007/s00330-012-2404-7 PMC3584153

[8] Rossi A Zoico E, *et al.*: Quantification of intermuscular adipose tissue in the erector spinae muscle by MRI: Agreement with histological evaluation. Obesity (Silver Spring). 2010; 18: 2379–2384. 20300085 10.1038/oby.2010.48 PMC5278643

[9] Smith AC Parrish TB, *et al.*: Muscle-fat MRI: 1.5 Tesla and 3.0 Tesla versus histology. Muscle Nerve. 2014; 50: 170–176. 24677256 10.1002/mus.24255 PMC6778690

[10] Yamada Y Yoshida T, *et al.*: The extracellular to intracellular water ratio in upper legs is negatively associated with skeletal muscle strength and gait speed in older people. J Gerontol A Biol Sci Med Sci.2017; 72: 293–298. 27422438 10.1093/gerona/glw125

[11] Taniguchi M Yamada Y, *et al.*: Increase in echo intensity and extracellular-to-intracellular water ratio is independently associated with muscle weakness in elderly women. Eur J Appl Physiol. 2017; 117: 2001–2007. 28755131 10.1007/s00421-017-3686-x

[12] Taniguchi M Yamada Y, *et al.*: Estimating thigh skeletal muscle volume using multi-frequency segmental-bioelectrical impedance analysis. J Physiol Anthropol. 2021; 40: 13. 34593041 10.1186/s40101-021-00263-z PMC8485471

[13] Pillen S Tak RO, *et al.*: Skeletal muscle ultrasound: Correlation between fibrous tissue and echo intensity. Ultrasound Med Biol. 2009; 35: 443–446. 19081667 10.1016/j.ultrasmedbio.2008.09.016

[14] Fukumoto Y Ikezoe T, *et al.*: Skeletal muscle quality assessed from echo intensity is associated with muscle strength of middle-aged and elderly persons. Eur J Appl Physiol. 2012; 112: 1519–1525. 21847576 10.1007/s00421-011-2099-5

[15] Taniguchi M Fukumoto Y, *et al.*: Quantity and quality of the lower extremity muscles in women with knee osteoarthritis. Ultrasound Med Biol. 2015; 41: 2567–2574. 26099784 10.1016/j.ultrasmedbio.2015.05.014

[16] Fukumoto Y Ikezoe T, *et al.*: Muscle mass and composition of the hip, thigh and abdominal muscles in women with and without hip osteoarthritis. Ultrasound Med Biol. 2012; 38: 1540–1545. 22749818 10.1016/j.ultrasmedbio.2012.04.016

[17] Arts IM Schelhaas HJ, *et al.*: Intramuscular fibrous tissue determines muscle echo intensity in amyotrophic lateral sclerosis. Muscle Nerve. 2012; 45: 449–450. 22334185 10.1002/mus.22254

[18] Fukumoto Y Taniguchi M, *et al.*: Influence of ultrasound focus depth on the association between echo intensity and intramuscular adipose tissue. Muscle Nerve. 2022; 66: 568–575. 35822539 10.1002/mus.27677

[19] Taniguchi M Fukumoto Y, *et al.*: Sitting vs. supine ultrasound measurements of the vastus medialis: Correlations with MRI measurements and age considerations. J Physiol Anthropol. 2023; 42: 14. 37454117 10.1186/s40101-023-00331-6 PMC10350276

[20] Fukumoto Y Ikezoe T, *et al.*: Age-related ultrasound changes in muscle quantity and quality in women. Ultrasound Med Biol. 2015; 41: 3013–3017. 26278633 10.1016/j.ultrasmedbio.2015.06.017

[21] Taniguchi M Fukumoto Y, *et al.*: Age-related changes in muscle thickness, echo intensity, and extracellular-to-intracellular water ratio. 23rd annual congress of the Japanese Society of Physical Therapy Fundamentals 2018; 46: Suppl.1

[22] Fukumoto Y Yamada Y, *et al.*: Association of physical activity with age-related changes in muscle echo intensity in older adults: A 4-year longitudinal study. J Appl Physiol (1985). 2018; 125: 1468–1474. 30113271 10.1152/japplphysiol.00317.2018

[23] Pedroso MG de Almeida AC, *et al.*: Fatty infiltration in the thigh muscles in knee osteoarthritis: A systematic review and meta-analysis. Rheumatol Int. 2019; 39: 627–635. 30852623 10.1007/s00296-019-04271-2

[24] Noehren B Kosmac K, *et al.*: Alterations in quadriceps muscle cellular and molecular properties in adults with moderate knee osteoarthritis. Osteoarthritis Cartilage. 2018; 26: 1359–1368. 29800621 10.1016/j.joca.2018.05.011 PMC7050996

[25] Taniguchi M Ikezoe T, *et al.*: Extracellular-to-intracellular water ratios are associated with functional disability levels in patients with knee osteoarthritis: Results from the Nagahama Study. Clin Rheumatol. 2021; 40: 2889–2896. 33486595 10.1007/s10067-021-05591-0

[26] Taniguchi M Fukumoto Y, *et al.*: Enhanced echo intensity in vastus medialis is associated with worsening of functional disabilities and symptoms in patients with knee osteoarthritis: A 3 years longitudinal study. Rheumatol Int.2023; 43: 953–960. 36394599 10.1007/s00296-022-05246-6 PMC9672570

[27] Taniguchi M Fukumoto Y, *et al.*: A higher intramuscular fat in vastus medialis is associated with functional disabilities and symptoms in early stage of knee osteoarthritis: A case-control study. Arthritis Res Ther. 2023; 25: 61. 37060080 10.1186/s13075-023-03048-0 PMC10103393

[28] Taniguchi M Fukumoto Y, *et al.*: Enhanced echo intensity and a higher extracellular water-to-intracellular water ratio are helpful clinical signs for detecting muscle degeneration in patients with knee osteoarthritis. Clin Rheumatol. 2021; 40: 4207–4215. 33999290 10.1007/s10067-021-05763-y

[29] Damas F Phillips SM, *et al.*: Resistance training-induced changes in integrated myofibrillar protein synthesis are related to hypertrophy only after attenuation of muscle damage. J Physiol. 2016; 594: 5209–5222. 27219125 10.1113/JP272472 PMC5023708

[30] Damas F Libardi CA, *et al.*: The development of skeletal muscle hypertrophy through resistance training: The role of muscle damage and muscle protein synthesis. Eur J Appl Physiol. 2018; 118: 485–500. 29282529 10.1007/s00421-017-3792-9

[31] Damas F Angleri V, *et al.*: Myofibrillar protein synthesis and muscle hypertrophy individualized responses to systematically changing resistance training variables in trained young men. J Appl Physiol (1985). 2019; 127: 806–815. 31268828 10.1152/japplphysiol.00350.2019

[32] Hirono T Ikezoe T, *et al.*: Relationship between muscle swelling and hypertrophy induced by resistance training. J Strength Cond Res. 2022; 36: 359–364. 31904714 10.1519/JSC.0000000000003478

[33] Chen TC Nosaka K: Responses of elbow flexors to two strenuous eccentric exercise bouts separated by three days. J Strength Cond Res. 2006; 20: 108–116. 16503669 10.1519/R-16634.1

[34] Maeo S Saito A, *et al.*: Localization of muscle damage within the quadriceps femoris induced by different types of eccentric exercises. Scand J Med Sci Sports. 2018; 28: 95–106. 28314055 10.1111/sms.12880

[35] Vandenborne K Walter G, *et al.*: Relationship between muscle T2* relaxation properties and metabolic state: A combined localized ^31^P-spectroscopy and ^1^H-imaging study. Eur J Appl Physiol. 2000; 82: 76–82. 10879446 10.1007/s004210050654

[36] Taniguchi M Yamada Y, *et al.*: Acute effect of multiple sets of fatiguing resistance exercise on muscle thickness, echo intensity, and extracellular-to-intracellular water ratio. Appl Physiol Nutr Metab. 2020; 45: 213–219. 31299164 10.1139/apnm-2018-0813

[37] Taniguchi M Hirono T, *et al.*: Assessment of edematous changes using three-dimensional body scanning and segmental-bioelectrical impedance spectroscopy. Lymphat Res Biol. 2021; 19: 524–530. 33605789 10.1089/lrb.2020.0087

